# Carotid plaque formation prediction model and validation: a case-control study

**DOI:** 10.3389/fcvm.2026.1798393

**Published:** 2026-04-24

**Authors:** JiLin Wu, XiaoPing Yang, Alimire Maimaitimin, Ayipali Aikemujiang, JunShi Zhang

**Affiliations:** 1 Department of Hypertension, The First Affiliated Hospital of Xinjiang Medical University, Urumqi, Xinjiang, China; 2 Health Management Center, The First Affiliated Hospital of Xinjiang Medical University, Urumqi, Xinjiang, China

**Keywords:** carotid plaque, LASSO, Logistic regression, Prediction model, Risk factors

## Abstract

**Background:**

Carotid plaque serves as an early window into atherosclerosis; however, more convenient tools for plaque risk stratification are currently lacking. This study aimed to investigate the risk factors for carotid plaque occurrence, establish a predictive model, and develop a risk assessment scale.

**Methods:**

A total of 12,391 individuals who underwent health examinations at the Physical Examination Center of the First Affiliated Hospital of Xinjiang Medical University between January 2024 and March 2025 were retrospectively enrolled. After applying inclusion and exclusion criteria, Least Absolute Shrinkage and Selection Operator (LASSO) regression was performed. The cohort was then randomly divided into a development set (*n* = 7,434) and a validation set (*n* = 4,957) to construct a binary multivariate logistic regression model.

**Results:**

In the multivariate regression model adjusted for confounding factors within the development set, female sex (OR = 0.59) and high-density lipoprotein cholesterol (HDL-c) >1.55 mmol/L (OR = 0.80) were associated with a reduced risk of plaque. Age 45–59 years (OR = 5.19), age ≥60 years (OR = 14.04), and smoking (OR = 1.37) were independently associated.

## 1 Introduction

Atherosclerotic cardiovascular disease (ASCVD) is the leading cause of death globally, accounting for over 40% of all deaths (1). In 2019, it was responsible for 18.6 million deaths worldwide, representing 45% of all non-communicable disease mortality (2).

Atherosclerosis is driven by the interplay of low-density lipoprotein (LDL) deposition, inflammation, and fibrosis. Oxidized LDL (OX-LDL) triggers the formation of foam cells, while smooth muscle cells synthesize the fibrous cap, which is a key determinant of plaque stability (3–5). Carotid artery plaques serve as a “window” to systemic atherosclerosis. Their presence can predict future cardiovascular events (6–8) and represents a critical stage in the progression from subclinical disease to acute ischemic events (9, 10).

Most previous studies have focused on constructing risk stratification scores for already established carotid plaques (11, 12). These approaches typically require further investigations such as magnetic resonance imaging (MRI) or contrast-enhanced ultrasound, imposing significant economic and psychological burdens on patients. Therefore, we propose performing risk stratification early, during routine health examinations in the general population, to enable better prevention and management of high-risk individuals. Recently, a cross-sectional study on cardiovascular risk factors developed a risk prediction model by constructing an internal validation set and identified factors such as age, education level, marital status, and current smoking history to predict the risk of carotid plaque formation in the general population (13). However, that study was a single-center investigation with a small sample size and lacked an external validation set. Furthermore, relying solely on a nomogram for risk stratification can prolong decision-making time and reduce implementation rates in scenarios requiring rapid risk assessment within seconds. This study utilizes a large-scale health examination cohort in China to investigate independent factors associated with carotid plaque formation. A risk stratification system for plaque development is constructed and validated, aiming to assist clinicians in identifying high-risk individuals for carotid plaques during the pre-symptomatic health stage for early intervention, and to enable refined risk stratification and dynamic management for those who have already developed plaques.

## 2 Materials and methods

### 2.1 Study population

**Figure 1:**
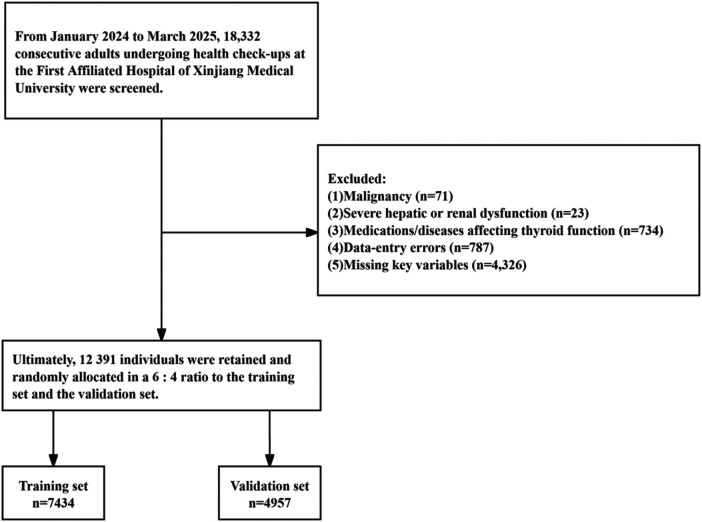
Flow chart of participant screening.

This study employed a retrospective cross-sectional design. Individuals who underwent health examinations at the Health Management Center of the First Affiliated Hospital of Xinjiang Medical University between January 2024 and March 2025 were selected. Subjects who received carotid artery ultrasound examinations, thyroid function tests, and related antibody assessments, and were aged between 18 and 80 years, were included in our overall population (*n* = 18,332). All participants completed questionnaires. To minimize the influence of thyroid disorders, the following exclusions were applied: a history of thyroid disease or thyroid surgery, long-term use of medications affecting thyroid function determination (*n* = 665); long-term use of hormones or a history of atrial fibrillation (including within 3–6 months post-radiofrequency ablation), oral amiodarone use, or use of lithium for mood disorders (*n* = 66); presence of severe hepatic or renal failure (*n* = 23); a history or current diagnosis of malignancy (*n* = 71) or autoimmune diseases (*n* = 11); and missing or outlier data in laboratory and/or imaging examinations (*n* = 5,105) (Figure 1). This study adhered to the principles of the Declaration of Helsinki and was approved by the Ethics Committee of the First Affiliated Hospital of Xinjiang Medical University (Approval No.: 240522-112).

## 3 Study indicators

### 3.1 Laboratory indicators

All study subjects fasted for 8 hours, and fasting venous blood was drawn the following morning. The following parameters were measured using a Roche cobas 8,000 fully automated biochemical analyzer: fasting blood glucose [GLU, mmol/L: reference range (3.9, 6.1) mmol/L], glycated hemoglobin [HbA1c, %: (4.8, 6.0)%], and five thyroid function indicators: thyroid-stimulating hormone [TSH, mIU/L: (0.27, 4.2) mIU/L], free thyroxine [FT4, pmol/L: (12, 22) pmol/L], free triiodothyronine [FT3, pmol/L: (3.1, 6.8) pmol/L], thyroglobulin antibody [TGAb, IU/mL: (0, 115) IU/mL], and thyroid peroxidase antibody [TPOAb, IU/mL: (0, 34) IU/mL]. Complete blood count was analyzed using an automated biochemical analyzer: white blood cell count [WBC, 10^9^/L: (3.50, 9.50)], neutrophil count [NE, 10^9^L: (1.8, 6.3)], lymphocyte count [LYMPH, 10^9^/L: (1.1, 3.2)], red blood cell count [RBC, 10^12^/L: adult males (4.30, 5.80), adult females (3.8, 5.1)], monocyte count [MONO, 10⁹/L: (0.10, 0.60)], and platelet count [PLT, 10^9^/L: (125, 350)]. Lipid profiles included: total cholesterol [TC, mmol/L: (2.8, 5.7) mmol/L], triglycerides [TG, mmol/L: (0.29, 1.83) mmol/L], low-density lipoprotein cholesterol [LDL-C, mmol/L: (2.7, 3.1) mmol/L], and high-density lipoprotein cholesterol [HDL-C, mmol/L: (1.16, 1.55) mmol/L]. Note: Reference ranges were based on the laboratory standards of the First Affiliated Hospital of Xinjiang Medical University, which were established according to domestic laboratory diagnostic criteria and calculated based on the hospital's actual clinical practice.

### 3.2 Imaging data

Carotid artery ultrasound examinations were performed using a Philips EPIQ 7C color Doppler ultrasound diagnostic system. Radiomic features were extracted focusing on four aspects of plaques: location, size, morphology, and echogenicity. Ultrasound scanning covered six carotid segments: the bilateral common carotid arteries, bilateral carotid bulbs, and bilateral proximal internal carotid arteries (first 1 cm segment) (14). Standardized measurement and description of carotid intima-media thickness (CIMT) and plaque size: Measurement of intima-media thickness (IMT) and plaques forms the basis for assessing carotid atherosclerotic lesions. An atherosclerotic plaque was defined when IMT was ≥1.5 mm, protruded into the vessel lumen or showed localized thickening exceeding 50% of the surrounding IMT (15).

Based on characteristics and health risk assessment of the Chinese adult population, body mass index (BMI, kg/m²), blood pressure (BP, mmHg), and waist circumference (WC, cm) were measured (16). Blood pressure was measured using a validated Omron HBP-9020 blood pressure monitor. The diagnosis of hypertension strictly followed the expert consensus criteria outlined in the Chinese Guidelines for the Prevention and Treatment of Hypertension (2024 Revision) (17). Waist circumference was measured using a soft tape measure placed horizontally around the abdomen at the level of the umbilicus, and the reading was recorded (18).

### 3.3 Questionnaire survey

The questionnaire covered age, education level, ethnicity, occupation, physical activity level (never exercise, exercise ≥3 times/week, exercise ≤3 times/week), smoking and alcohol consumption status (never, former smoker/drinker, current smoker/drinker), as well as family history of cardiovascular diseases, thyroid diseases, and other conditions, and personal past medical history.

### 3.4 Study outcome

The study subjects were adult examinees who completed the relevant examinations. The primary diagnostic basis was neck vascular color Doppler ultrasound and the five thyroid function tests. The study outcome was defined as the presence or absence of carotid artery plaques.

## 4 Methods: statistical analysis

### 4.1 Model development and evaluation

Data analysis was performed using IBM SPSS Statistics 27. Data organization and statistical analysis were conducted with R version 4.3.3 and DMasS (version 1.5.0). The following R packages were utilized: glmnet 4.1.7, rms 4.6.0, Resource Selection (0.3–5), pROC (1.18.0), and rmda.

#### 4.1.1 Baseline data analysis

All continuous variables were first subjected to the Kolmogorov–Smirnov test, and the results indicated that none followed a normal distribution (*P* < 0.05). Therefore, they were described using the median (interquartile range) [M(IQR)], and between-group comparisons were performed using the Wilcoxon Mann–Whitney test. Categorical variables were expressed as n (%), and between-group comparisons were conducted using the *χ*^2^ test. All tests were two-sided, with a significance level of *α* = 0.05. A *P*-value < 0.05 was considered statistically significant.

#### 4.1.2 Prediction model development

The total dataset (*n* = 3,000) was randomly subjected to LASSO regression for variable screening. Subsequently, the data were stratified and randomly split into a development set and a validation set at a 6:4 ratio. The development set comprised 7,434 cases, and the validation set comprised 4,957 cases. Within the development set, univariate logistic regression was first performed. Variables with *P* < 0.05 were then included in a multivariate logistic regression model using a bidirectional stepwise selection method. Based on the multivariate results, a nomogram and calibration curves were constructed using R. The receiver operating characteristic (ROC) curve was plotted for the model, and the area under the curve (AUC) was calculated. The robustness of the model was verified by comparing the AUC, F1 score, and Brier score between the development and validation sets. Based on the results of the multivariate logistic regression analysis from the development set, a clinical scoring table for individualized risk assessment was constructed. Binary logistic regression verification demonstrated that this risk stratification system exhibited a clear gradient risk association in both the development and validation sets.

#### 4.1.3 Detailed methodology for prediction model development and performance validation

First, baseline data analysis for the total of 12,391 cases was performed in SPSS. For numerical variables, normality was tested using the Kolmogorov–Smirnov test, where a *p*-value > 0.05 indicates normality. It was found that all variables deviated from a normal distribution. Therefore, non-parametric tests (Mann–Whitney U test) were further conducted to preliminarily observe the distribution differences of 22 clinical and biochemical indicators between the two groups (with and without carotid artery plaque). For categorical variables, frequency distributions were used, and these variables were analyzed using the chi-square test (cross-tabulation).

**Figure 2:**
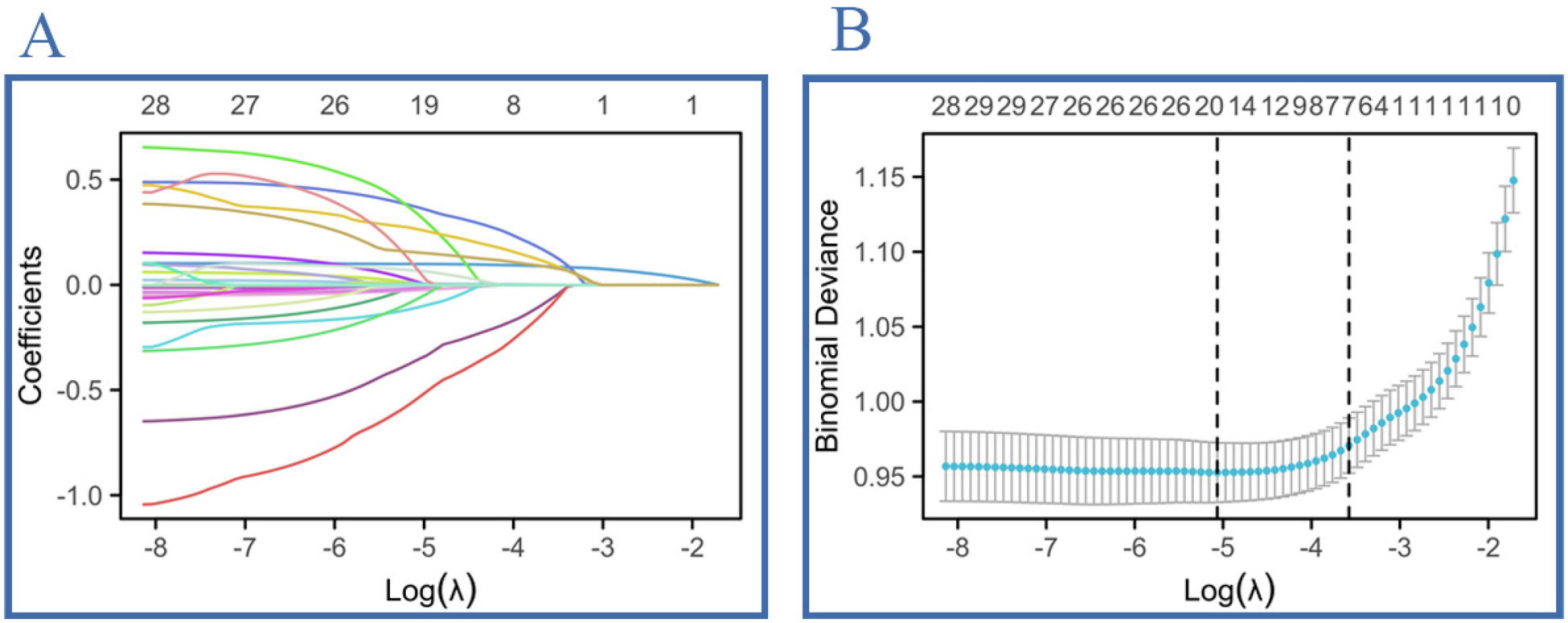
**(A)** ROC curves: training AUC 0.79 (0.78–0.80) and validation AUC 0.80 (0.78–0.81). **(B)** Nomogram converting covariates to points for individual plaque probability. **(C)** Decision-curve analysis, training set. **(D)** Decision-curve analysis, validation set. **(E)** Calibration plot, training set; Hosmer-Lemeshow *P* = 0.468. **(F)** Calibration plot, validation set; Hosmer-Lemeshow *P* = 0.091.

A total of 29 variables were initially included in this study. To avoid overfitting, LASSO regression was first employed for preliminary variable screening. LASSO regression, by imposing a penalty on regression coefficients (L1 regularization), can shrink the coefficients of unimportant variables to zero, thereby achieving automatic variable selection (19). This screening process ultimately yielded 26 non-zero variables, selected based on the “*λ*.min” criterion. The screened variables were then dummy-coded and randomly divided into development and validation sets, with stratification to ensure consistent proportions of the outcome event. Within the development set, univariate analysis followed by binary multivariate logistic regression analysis (bidirectional stepwise regression) was performed to identify significant variables (*P* < 0.05) and construct the logistic regression model. This model was used to generate the receiver operating characteristic (ROC) curve analysis (Figure 2A), an indicator commonly used to evaluate the discriminative ability of diagnostic or predictive models (20). The ROC curve was also used to assess predictive diagnostic performance. Decision curve analysis (DCA) curves (Figures 2C,D) were generated by calculating the net benefit of the model across different threshold probabilities to determine its potential clinical utility (21). Calibration curves were plotted to evaluate the model's predictive accuracy (Figures 2E,F), and quantitative validation was performed using the Hosmer-Lemeshow goodness-of-fit test. A nomogram was also constructed, converting the regression coefficients of each predictor variable into an intuitive linear scoring scale, which can be used to calculate the individualized risk of carotid artery plaque occurrence (Figure 2B). Based on the multivariate logistic regression coefficients (β), integer scores were calculated using the formula (β/|βmin|) to construct a clinical scoring table for individualized risk assessment (see Table 1). Subsequently, this scoring table was applied to calculate the total risk score for each individual in both the development and validation sets. Risk stratification boundaries were predefined before model construction according to the conventional low-, medium-, and high-risk tripartite stratification. Finally, binary logistic regression verification was performed on the data segmented by these risk levels to test for statistical significance and clinical gradient differences between adjacent risk categories.

**Table 1:** Risk scoring table for carotid plaque occurrence in adults (applied in both training and validation datasets).

Characteristics	Categories	Points
Sex	Female	−3
Age	45–59years	9
	>60years	15
Smoking	Yes	2
Hypertension	Yes	3
Coronay Heart Disease	Yes	4
Diabetes	Yes	2
TSH	>4.2 mIU/mL	1
HDL	>1.55 mmol/L	−1
TC	>5.7 mmol/L	2
NE	>6.310⁹/L	2
RBC	Male: <4.30 × 10^12^/L; Female: <3.8 × 10^12^/L	4
HbA1C	<4.8%	−5

Total score = sum of points for present characteristics; higher scores reflect greater predicted probability. Performance and clinical utility need external validation.

## 5 Results

### 5.1 Analysis of baseline data differences based on carotid plaque status

The results of the non-parametric tests for each variable are shown in Table 2. Significant differences (*p* < 0.05) were observed between the two groups in 19 indicators, including age and BMI, while no significant differences were found in the remaining indicators. Based on the *Z*-values obtained from the Mann–Whitney *U*-test, the plaque group exhibited significantly higher values for indicators such as BMI, blood pressure, and blood glucose compared to the non-plaque group, suggesting these factors may be risk factors for carotid plaque formation. The results of the chi-square test indicated that samples with different carotid plaque status showed significant differences in the aforementioned categorical indicators (*p* < 0.05).

**Table 2:** Baseline characteristics by group.

Variable	Non-plaque group (*n* = 7,345)	Plaque group (*n* = 5,046)	Z/*χ*^2^	*p*-value
Age (years)	40.00 (34.00, 49.00)	53.00 (48.00, 59.00)	−56.233	<0.001
BMI (kg/m^2^)	25.48 (23.20, 27.90)	25.98 (24.00, 28.20)	−8.377	<0.001
SBP (mmHg)	122.00 (113.00, 133.00)	128.00 (117.00, 140.00)	−19.511	<0.001
DBP (mmHg)	76.00 (69.00, 84.00)	79.00 (72.00, 86.00)	−14.092	<0.001
WC (cm)	88.00 (80.00, 95.00)	90.00 (85.00, 97.00)	−15.139	<0.001
Thyroid Function				
TSH (mIU/L)	2.00 (1.40, 2.80)	2.15 (1.50, 3.10)	−7.575	<0.001
FT4 (pmol/L)	16.90 (15.40, 18.60)	16.60 (15.10, 18.30)	−6.539	<0.001
FT3 (pmol/L)	5.20 (4.80, 5.60)	5.07 (4.70, 5.50)	−10.103	<0.001
Thyroid Antibodies				
TGAb (IU/mL)	17.90 (15.90, 20.40)	17.90 (15.70, 20.90)	−0.137	0.891
TPOAb (IU/mL)	11.80 (9.00, 15.80)	11.65 (9.00, 16.00)	−0.469	0.639
Glucose Metabolism				
HbA1c (%)	5.54 (5.30, 5.80)	5.78 (5.50, 6.20)	−30.187	<0.001
GLU (mmol/L)	4.83 (4.50, 5.20)	5.06 (4.70, 5.60)	−22.083	<0.001
Lipid Profile				
TC (mmol/L)	4.57 (4.00, 5.20)	4.73 (4.10, 5.40)	−6.725	<0.001
TG (mmol/L)	1.40 (0.90, 2.10)	1.55 (1.10, 2.20)	−9.999	<0.001
HDL (mmol/L)	1.19 (1.00, 1.40)	1.17 (1.00, 1.40)	−4.508	<0.001
LDL (mmol/L)	2.85 (2.40, 3.40)	2.94 (2.30, 3.50)	−3.865	<0.001
Blood Cell Counts				
WBC (×10^9^/L)	5.90 (5.00, 7.00)	5.94 (5.00, 7.10)	−1.525	0.127
NE (×10^9^/L)	3.32 (2.70, 4.10)	3.38 (2.70, 4.20)	−3.104	0.002
LYMPH (×10^9^/L)	1.92 (1.60, 2.30)	1.87 (1.50, 2.30)	−4.239	<0.001
MONO (×10^9^/L)	0.41 (0.30, 0.50)	0.43 (0.30, 0.50)	−7.000	<0.001
PLT (×10^9^/L)	243.00 (211.00, 280.00)	231.00 (199.00, 267.00)	−12.914	<0.001
RBC (×10^12^/L)	5.07 (4.70, 5.40)	4.99 (4.70, 5.30)	−6.292	<0.001
Sex, *n* (%)			80.025	<0.001
Female	5,330 (72.57)	4,017 (79.61)		
Male	2,015 (27.43)	1,029 (20.39)		
Smoking, *n* (%)			104.890	<0.001
No	5,113 (69.61)	3,065 (60.74)		
Yes	2,232 (30.39)	1,981 (39.26)		
Drinking, *n* (%)			72.210	<0.001
No	2,059 (28.03)	1,777 (35.22)		
Yes	5,286 (71.97)	3,269 (64.78)		
Exercise, *n* (%)			301.950	<0.001
Never	1,372 (18.68)	691 (13.69)		
Occasional	4,118 (56.07)	2,344 (46.45)		
Regular	1,855 (25.26)	2,011 (39.85)		
Hypertension			911.174	<0.001
0	6,730 (91.63)	3,583 (71.01)		
1	615 (8.37)	1,463 (28.99)		
Coronary Heart Disease			433.174	<0.001
0	7,244 (98.62)	4,573 (90.63)		
1	101 (1.38)	473 (9.37)		
Diabetes			400.158	<0.001
0	7,095 (96.60)	4,395 (87.10)		
1	250 (3.40)	651 (12.90)		

Data are presented as n (%) or median [IQR]. Between-group comparisons used χ^2^-test (categorical) or non-parametric test (continuous). *P* < 0.05 was considered statistically significant.

BMI, body-mass index; DBP, diastolic blood pressure; FT3, free triiodothyronine; FT4, free thyroxine; GLU, fasting glucose; HbA1c, glycated haemoglobin; HDL-C, high-density-lipoprotein cholesterol; LDL-C, low-density-lipoprotein cholesterol; LYMPH, lymphocyte count; MONO, monocyte count; NE, neutrophil count; PLT, platelet count; RBC, red-blood-cell count; SBP, systolic blood pressure; TC, total cholesterol; TG, triglycerides; TGAb, thyroglobulin antibody; TPOAb, thyroid-peroxidase antibody; TSH, thyroid-stimulating hormone; WC, waist circumference; WBC, white-blood-cell count.

### 5.2 Comparison of the development and test sets for carotid plaque presence

**Figure 3:**
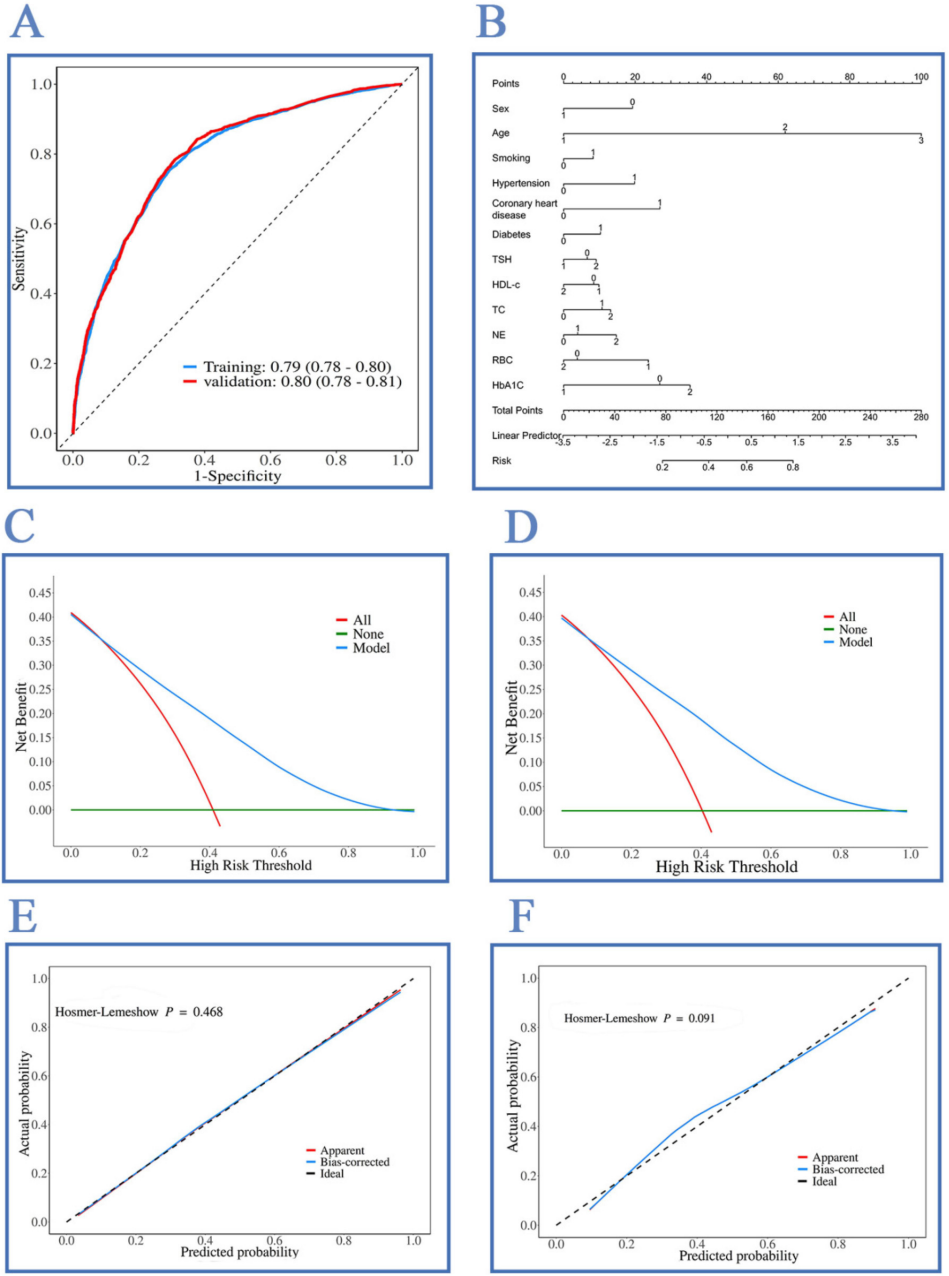
**(A)** Lasso coefficient–profile plot: coefficient magnitude for each variable across the log(*λ*) spectrum; 26 predictors retained at the value indicated. **(B)** Ten-fold cross-validation deviance path: minimum binomial deviance identifies the optimal *λ* yielding the final 26-variable signature.

In this study, the variables selected via Lasso regression from the final cohort of 12,391 participants were subjected to dummy variable assignment. Details of the dummy variable assignment are provided in Table 3. The variable trajectory and selection process of the Lasso regression are shown in Figure 3. As indicated by the results in Table 4, no significant differences were observed for any variable between the development set and the test set (*P* > 0.05). In Table 5, the distributions of all demographic characteristics, lifestyle factors, medical history, and laboratory indicators showed no statistically significant differences between the development set and the validation set (all *P* > 0.05).

**Table 3:** Dummy variable assignment table for each variable.

Variables	Dummy Variable Assignment Rules
Gender	Male = 0; Female = 1
Age	Youth (18–44) = 1,;Middle-aged (45–59) = 2; Elderly (>60) = 3
Systolic Blood Pressure (SBP, mmHg)	[90, 140) = 0; ≥140 = 1
Diastolic Blood Pressure (DBP, mmHg)	[60, 89)= 0; ≥90 = 1
Coronary Heart Disease	No = 0; Yes = 1
Hypertension (HNT)	No = 0; Yes = 1
Diabetes	No = 0; Yes = 1
Severe hepatic or renal impairment	No = 0; Yes = 1
Long-term corticosteroid therapy	No = 0; Yes = 1
Autoimmune disease	No = 0; Yes = 1
Malignancy	No = 0; Yes = 1
Depression (lithium)	No = 0; Yes = 1
Smoking	No = 0; Yes = 1
Drinking	No = 0; Yes = 1
Carotid Artery Plaque	No = 0; Yes = 1
Red Blood Cell (RBC, 10¹²/L)	Male: [4.30, 5.80] = 0; <4.30 = 1; >5.80 = 2 Female: [3.8–5.1] = 0; <3.8 = 1; >5.1 = 2
Platelet (PLT, 10⁹/L)	[125, 350] = 0; <125 = 1; >350 = 2
Monocyte (MONO, 10⁹/L)	[0.10, 0.60] = 0; >0.60 = 1
Lymphocyte (LYMPH, 10⁹/L)	[1.1, 3.2] = 0; < 1.1 = 1; >3.2 = 2
Neutrophil (NE, 10⁹/L)	[1.8, 6.3] = 0; < 1.8 = 1; > 6.3 = 2
White Blood Cell (WBC, 10⁹/L)	[3.50, 9.50] = 0; <3.50 = 1; >9.50 = 2
Glucose (GLU, mmol/L)	[3.9, 6.1] = 0; <3.9 = 1; >6.1 = 2
Glycated Hemoglobin (HbA1c, %)	[4.8, 6.0] = 0; <4.8 = 1; >6.0 = 2
Triglycerides (TG, mmol/L)	[0.29, 1.83] = 0; >1.83 = 1
Total Cholesterol (TC, mmol/L)	[2.8, 5.7] = 0; <2.8 = 1; >5.7 = 2
Low-Density Lipoprotein (LDL-c, mmol/L)	[2.7, 3.1] = 0; <2.7 = 1; >3.1 = 2
High-Density Lipoprotein (HDL-c, mmol/L)	[1.16, 1.55] = 0; <1.16 = 1; >1.55 = 2
Thyroperoxidase Antibody (TPOAb, IU/mL)	[0, 34] = 0; >34 = 1
Thyroglobulin Antibody (TGAb, IU/mL)	[0, 115] = 0; >115 = 1
Free Triiodothyronine (FT3, pmol/L)	[3.1, 6.8] = 0; <3.1 = 1; >6.8 = 2
Free Thyroxine (FT4, pmol/L)	[12, 22] = 0; <12 = 1; >22 = 2
Thyroid-Stimulating Hormone (TSH, mIU/mL)	[0.27, 4.2] = 0; <0.27 = 1; >4.2 = 2
Waist Circumference (WC, cm)	Men: <90 = 0, ≥90 = 1; Women: <85 = 0,≥85 = 1
Body Mass Index (BMI)	<18.5 = 1 (Underweight); [18.5–23.9] = 0 (Normal); [24.0–27.9] = 2 (Overweight); ≥28（Obesity) = 3;
Weekly Exercise Frequency	No Exercise = 0; <3 times/week = 1; ≥3 times/week = 2

Categorical variables were dummy-coded: binary as 0 (absent/normal) and 1 (present/abnormal); ordinal variables as specified. Continuous variables were categorised using clinical cut-offs.

**Table 4:** Comparison of risk-Stratum assignment between carotid-plaque and No-plaque groups in the training Set.

Variables	Total (*n* = 7,434)	Non-Plaque Group (*n* = 4,389)	Plaque Group1 (*n* = 3,045)	*t*/*Z*/*χ*²	*P*
Sex, *n* (%)				51.53	<0.001
Male	5,600 (75.33)	3,175 (72.34)	2,425 (79.64)		
Female	1,834 (24.67)	1,214 (27.66)	620 (20.36)		
Age, *n* (%)				1,539.37	<0.001
18–44	3,272 (44.01)	2,713 (61.81)	559 (18.36)		
45–59	3,314 (44.58)	1,497 (34.11)	1,817 (59.67)		
>60	848 (11.41)	179 (4.08)	669 (21.97)		
Smoking, *n* (%)				84.00	<0.001
No	4,905 (65.98)	3,080 (70.18)	1,825 (59.93)		
Yes	2,529 (34.02)	1,309 (29.82)	1,220 (40.07)		
Drinking, *n* (%)				36.14	<0.001
No	2,312 (31.10)	1,247 (28.41)	1,065 (34.98)		
Yes	5,122 (68.90)	3,142 (71.59)	1,980 (65.02)		
Exercise, *n* (%)				175.03	<0.001
No Exercise	1,276 (17.16)	841 (19.16)	435 (14.29)		
<3 times/week (Occasional exercise)	3,830 (51.52)	2,432 (55.41)	1,398 (45.91)		
≥3 times/week (Regular exercise)	2,328 (31.32)	1,116 (25.43)	1,212 (39.80)		
Hypertension, *n* (%)				508.86	<0.001
No	6,193 (83.31)	4,013 (91.43)	2,180 (71.59)		
Yes	1,241 (16.69)	376 (8.57)	865 (28.41)		
Coronay Heart Disease, *n* (%)				239.00	<0.001
No	7,096 (95.45)	4,326 (98.56)	2,770 (90.97)		
Yes	338 (4.55)	63 (1.44)	275 (9.03)		
Diabetes, *n* (%)				218.90	<0.001
No	6,914 (93.01)	4,242 (96.65)	2,672 (87.75)		
Yes	520 (6.99)	147 (3.35)	373 (12.25)		
SBP, *n* (%)				140.77	<0.001
[90, 139]	6,017 (80.94)	3,750 (85.44)	2,267 (74.45)		
≥140	1,417 (19.06)	639 (14.56)	778 (25.55)		
DBP, *n* (%)				36.55	<0.001
[60, 89]	6,306 (84.83)	3,815 (86.92)	2,491 (81.81)		
≥90	1,128 (15.17)	574 (13.08)	554 (18.19)		
WC, *n* (%)				117.35	<0.001
Male: <90 Female: <85	3,435 (46.21)	2,257 (51.42)	1,178 (38.69)		
Male: ≥90 Female: ≥85	3,999 (53.79)	2,132 (48.58)	1,867 (61.31)		
TSH, *n* (%)				39.38	<0.001
[0.27, 4.2]	6,656 (89.53)	4,000 (91.14)	2,656 (87.22)		
<0.27	71 (0.96)	48 (1.09)	23 (0.76)		
>4.2	707 (9.51)	341 (7.77)	366 (12.02)		
FT4, *n* (%)				4.63	0.099
[12, 22]	7,186 (96.66)	4,229 (96.35)	2,957 (97.11)		
<12	78 (1.05)	46 (1.05)	32 (1.05)		
>22	170 (2.29)	114 (2.60)	56 (1.84)		
FT3, *n* (%)				-	0.034
[3.1, 6.8]	7,335 (98.67)	4,319 (98.41)	3,016 (99.05)		
<3.1	7 (0.09)	4 (0.09)	3 (0.10)		
>6.8	92 (1.24)	66 (1.50)	26 (0.85)		
TGAb, *n* (%)				2.41	0.121
[0, 115]	6,897 (92.78)	4,089 (93.16)	2,808 (92.22)		
>115	537 (7.22)	300 (6.84)	237 (7.78)		
TPOAb, *n* (%)				5.95	0.015
[0, 34]	6,799 (91.46)	4,043 (92.12)	2,756 (90.51)		
>34	635 (8.54)	346 (7.88)	289 (9.49)		
HDL, *n* (%)				16.05	<0.001
[1.16, 1.55]	2,979 (40.07)	1,740 (39.64)	1,239 (40.69)		
<1.16	3,398 (45.71)	1,966 (44.79)	1,432 (47.03)		
>1.55	1,057 (14.22)	683 (15.56)	374 (12.28)		
TC, *n* (%)				67.06	<0.001
[2.8, 5.7]	6,287 (84.57)	3,830 (87.26)	2,457 (80.69)		
<2.8	105 (1.41)	38 (0.87)	67 (2.20)		
>5.7	1,042 (14.02)	521 (11.87)	521 (17.11)		
TG, *n* (%)				29.32	<0.001
[0.29, 1.83]	4,816 (64.78)	2,953 (67.28)	1,863 (61.18)		
>1.83	2,618 (35.22)	1,436 (32.72)	1,182 (38.82)		
GLU, *n* (%)				161.05	<0.001
[3.9, 6.1]	6,623 (89.09)	4,066 (92.64)	2,557 (83.97)		
<3.9	56 (0.75)	39 (0.89)	17 (0.56)		
>6.1	755 (10.16)	284 (6.47)	471 (15.47)		
NE, *n* (%)				8.81	0.012
[1.8, 6.3]	7,041 (94.71)	4,166 (94.92)	2,875 (94.42)		
<1.8	221 (2.97)	139 (3.17)	82 (2.69)		
>6.3	172 (2.31)	84 (1.91)	88 (2.89)		
LYMPH, *n* (%)				5.29	0.071
[1.1, 3.2]	6,948 (93.46)	4,122 (93.92)	2,826 (92.81)		
<1.1	233 (3.13)	121 (2.76)	112 (3.68)		
>3.2	253 (3.40)	146 (3.33)	107 (3.51)		
MONO, *n* (%)				20.55	<0.001
[0.10, 0.60]	6,587 (88.61)	3,950 (90.00)	2,637 (86.60)		
>0.60	847 (11.39)	439 (10.00)	408 (13.40)		
RBC, *n* (%)				35.15	<0.001
Male: [4.3, 5.8] Female: [3.8–5.1]	6,913 (92.99)	4,083 (93.03)	2,830 (92.94)		
Male: <4.30 Female: <3.8	103 (1.39)	34 (0.77)	69 (2.27)		
Male: >5.80 Female: >5.1	418 (5.62)	272 (6.20)	146 (4.79)		
PLT, *n* (%)				9.72	0.008
[125, 350]	7,137 (96.00)	4,197 (95.63)	2,940 (96.55)		
<125	47 (0.63)	23 (0.52)	24 (0.79)		
>350	250 (3.36)	169 (3.85)	81 (2.66)		
HbA1C, *n* (%)				344.11	<0.001
[4.8, 6.0]	5,881 (79.11)	3,765 (85.78)	2,116 (69.49)		
<4.8	65 (0.87)	55 (1.25)	10 (0.33)		
>6.0	1,488 (20.02)	569 (12.96)	919 (30.18)		

Values are *n* (%). *χ*^2^-test for between-group comparisons.

**Table 5:** Comparison of dummy Variable allocation in the training and validation sets of the examined population.

Variables	Total (*n* = 12,391)	Validation (*n* = 4,957)	Training (*n* = 7,434)	χ²	*P*
Carotid Artery Plaque, *n* (%)				0.43	0.51
No	7,345 (59.28)	2,956 (59.63)	4,389 (59.04)		
Yes	5,046 (40.72)	2,001 (40.37)	3,045 (40.96)		
Sex, *n* (%)				0.11	0.741
Male	9,347 (75.43)	3,747 (75.59)	5,600 (75.33)		
Female	3,044 (24.57)	1,210 (24.41)	1,834 (24.67)		
Age, *n* (%)				0.42	0.812
18–44	5,460 (44.06)	2,188 (44.14)	3,272 (44.01)		
45–59	5,536 (44.68)	2,222 (44.83)	3,314 (44.58)		
>60	1,395 (11.26)	547 (11.03)	848 (11.41)		
Smoking, *n* (%)				0	0.957
No	8,178 (66.00)	3,273 (66.03)	4,905 (65.98)		
Yes	4,213 (34.00)	1,684 (33.97)	2,529 (34.02)		
Drinking, *n* (%)				0.18	0.675
No	3,836 (30.96)	1,524 (30.74)	2,312 (31.10)		
Yes	8,555 (69.04)	3,433 (69.26)	5,122 (68.90)		
Exercise, *n* (%)				4.46	0.108
No Exercise	2,063 (16.65)	787 (15.88)	1,276 (17.16)		
<3 times/week (Occasional exercise)	6,462 (52.15)	2,632 (53.10)	3,830 (51.52)		
≥3 times/week (Regular exercise)	3,866 (31.20)	1,538 (31.03)	2,328 (31.32)		
Hypertension, *n* (%)				0.08	0.78
No	10,313 (83.23)	4,120 (83.11)	6,193 (83.31)		
Yes	2,078 (16.77)	837 (16.89)	1,241 (16.69)		
Coronay Heart Disease, *n* (%)				0.31	0.578
No	11,817 (95.37)	4,721 (95.24)	7,096 (95.45)		
Yes	574 (4.63)	236 (4.76)	338 (4.55)		
Diabetes, *n* (%)				2.11	0.147
No	11,490 (92.73)	4,576 (92.31)	6,914 (93.01)		
Yes	901 (7.27)	381 (7.69)	520 (6.99)		
SBP, *n* (%)				0.2	0.656
[90, 139]	10,045 (81.07)	4,028 (81.26)	6,017 (80.94)		
≥140	2,346 (18.93)	929 (18.74)	1,417 (19.06)		
DBP, *n* (%)				1.39	0.238
[60, 89]	10,549 (85.13)	4,243 (85.60)	6,306 (84.83)		
≥90	1,842 (14.87)	714 (14.40)	1,128 (15.17)		
WC, *n* (%)				0.1	0.75
Male: <90 Female: <85	5,711 (46.09)	2,276 (45.91)	3,435 (46.21)		
Male: ≥90 Female: ≥85	6,680 (53.91)	2,681 (54.09)	3,999 (53.79)		
TSH, *n* (%)				0.3	0.859
[0.27, 4.2]	11,081 (89.43)	4,425 (89.27)	6,656 (89.53)		
<0.27	122 (0.98)	51 (1.03)	71 (0.96)		
>4.2	1,188 (9.59)	481 (9.70)	707 (9.51)		
FT4, *n* (%)				0.75	0.686
[12, 22]	11,977 (96.66)	4,791 (96.65)	7,186 (96.66)		
<12	137 (1.11)	59 (1.19)	78 (1.05)		
>22	277 (2.24)	107 (2.16)	170 (2.29)		
FT3, *n* (%)				0.13	0.939
[3.1, 6.8]	12,229 (98.69)	4,894 (98.73)	7,335 (98.67)		
<3.1	12 (0.10)	5 (0.10)	7 (0.09)		
>6.8	150 (1.21)	58 (1.17)	92 (1.24)		
TGAb, *n* (%)				0.36	0.547
[0, 115]	11,510 (92.89)	4,613 (93.06)	6,897 (92.78)		
>115	881 (7.11)	344 (6.94)	537 (7.22)		
TPOAb, *n* (%)				0.17	0.68
[0, 34]	11,343 (91.54)	4,544 (91.67)	6,799 (91.46)		
>34	1,048 (8.46)	413 (8.33)	635 (8.54)		
HDL, *n* (%)				0.33	0.849
[1.16, 1.55]	4,943 (39.89)	1,964 (39.62)	2,979 (40.07)		
<1.16	5,689 (45.91)	2,291 (46.22)	3,398 (45.71)		
>1.55	1,759 (14.20)	702 (14.16)	1,057 (14.22)		
TC, *n* (%)				3	0.223
[2.8, 5.7]	10,519 (84.89)	4,232 (85.37)	6,287 (84.57)		
<2.8	184 (1.48)	79 (1.59)	105 (1.41)		
>5.7	1,688 (13.62)	646 (13.03)	1,042 (14.02)		
TG, *n* (%)				3.66	0.056
[0.29, 1.83]	8,110 (65.45)	3,294 (66.45)	4,816 (64.78)		
>1.83	4,281 (34.55)	1,663 (33.55)	2,618 (35.22)		
GLU, *n* (%)				3.96	0.138
[3.9, 6.1]	11,014 (88.89)	4,391 (88.58)	6,623 (89.09)		
<3.9	110 (0.89)	54 (1.09)	56 (0.75)		
>6.1	1,267 (10.23)	512 (10.33)	755 (10.16)		
NE, *n* (%)				4.52	0.104
[1.8, 6.3]	11,692 (94.36)	4,651 (93.83)	7,041 (94.71)		
<1.8	389 (3.14)	168 (3.39)	221 (2.97)		
>6.3					
LYMPH, *n* (%)				2.79	0.248
[1.1, 3.2]					
<1.1	411 (3.32)	178 (3.59)	233 (3.13)		
>3.2					
MONO, *n* (%)				0.14	0.708
[0.10, 0.60]	10,990 (88.69)	4,403 (88.82)	6,587 (88.61)		
>0.60	1,401 (11.31)	554 (11.18)	847 (11.39)		
RBC, *n* (%)				0.59	0.745
Male: [4.3, 5.8] Female: [3.8–5.1]	11,534 (93.08)	4,621 (93.22)	6,913 (92.99)		
Male: <4.30 Female: <3.8	175 (1.41)	72 (1.45)	103 (1.39)		
Male: >5.80 Female: >5.1	682 (5.50)	264 (5.33)	418 (5.62)		
PLT, *n* (%)				0.46	0.793
[125, 350]	11,900 (96.04)	4,763 (96.09)	7,137 (96.00)		
<125	82 (0.66)	35 (0.71)	47 (0.63)		
>350	409 (3.30)	159 (3.21)	250 (3.36)		
HbA1C, *n* (%)				0.47	0.792
[4.8, 6.0]	9,785 (78.97)	3,904 (78.76)	5,881 (79.11)		
<4.8	105 (0.85)	40 (0.81)	65 (0.87)		
>6.0	2,501 (20.18)	1,013 (20.44)	1,488 (20.02)		

χ^2^, Chi-square test.

### 5.3 Results of univariate and multivariate logistic regression analyses in the development set

The results of the binary logistic regression model (Table 6) indicated that female sex, age [45–59 years (middle-aged) OR = 5.19, ≥60 years (elderly) OR = 14.04], smoking, a history of hypertension, coronary heart disease, or diabetes, TSH > 4.2 mIU/L (OR = 1.20), HDL-C > 1.55 mmol/L (OR = 0.80), TC > 5.7 mmol/L (OR = 1.42), NE >6.3 × 10^9^/L (OR = 1.47), RBC (Male: <4.30; Female: <3.8), and HbA1c < 4.8% (OR = 0.37) were all characteristic factors (*p* < 0.05).

**Table 6:** Presentation of single-factor and multiple-factor logistic regression results in the training set.

Variables	Univariate analysis			Multivariate analysis		
	*β*	*P*	OR (95%CI)	β	*P*	OR (95%CI)
Sex						
Male			1.00 (Reference)			1.00 (Reference)
Female	−0.4	<0.001	0.67 (0.60–0.75)	−0.53	<0.001	0.59 (0.51–0.69)
Age						
18–44			1.00 (Reference)			1.00 (Reference)
45–59	1.77	<0.001	5.89 (5.26–6.60)	1.65	<0.001	5.19 (4.59–5.86)
>60	2.9	<0.001	18.14 (15.02–21.90)	2.64	<0.001	14.04 (11.33–17.41)
Smoking						
No			1.00 (Reference)			1.00 (Reference)
Yes	0.45	<0.001	1.57 (1.43–1.73)	0.31	<0.001	1.37 (1.21–1.55)
Drinking						
No			1.00 (Reference)			1.00 (Reference)
Yes	−0.3	<0.001	0.74 (0.67–0.81)	−0.12	0.068	0.89 (0.78–1.01)
Exercise						
No Exercise			1.00 (Reference)			
<3 times/week (Occasional exercise)	0.11	0.12	1.11 (0.97–1.27)			
≥3 times/week (Regular exercise)	0.74	<0.001	2.10 (1.82–2.42)			
Hypertension						
No			1.00 (Reference)			1.00 (Reference)
Yes	1.44	<0.001	4.23 (3.71–4.83)	0.5	<0.001	1.65 (1.41–1.92)
Coronay Heart Disease						
No			1.00 (Reference)			1.00 (Reference)
Yes	1.92	<0.001	6.82 (5.16–9.00)	0.66	<0.001	1.94 (1.43–2.64)
Diabetes						
No			1.00 (Reference)			1.00 (Reference)
Yes	1.39	<0.001	4.03 (3.31–4.91)	0.32	0.011	1.37 (1.08–1.75)
SBP						
[90, 139]			1.00 (Reference)			
≥140	0.7	<0.001	2.01 (1.79–2.26)			
DBP						
[60, 89]			1.00 (Reference)			
≥90	0.39	<0.001	1.48 (1.30–1.68)			
WC						
Male: <90 Female: <85			1.00 (Reference)			
Male: ≥90 Female: ≥85	0.52	<0.001	1.68 (1.53–1.84)			
TSH						
[0.27, 4.2]			1.00 (Reference)			1.00 (Reference)
<0.27	−0.33	0.2	0.72 (0.44–1.19)	−0.26	0.371	0.77 (0.44–1.36)
>4.2	0.48	<0.001	1.62 (1.38–1.89)	0.18	0.045	1.20 (1.01–1.44)
FT4						
[12, 22]			1.00 (Reference)			
<12	−0.01	0.982	0.99 (0.63–1.57)			
>22	−0.35	0.032	0.70 (0.51–0.97)			
FT3						
[3.1, 6.8]			1.00 (Reference)			
<3.1	0.07	0.926	1.07 (0.24–4.80)			
>6.8	−0.57	0.014	0.56 (0.36–0.89)			
TGAb						
[0, 115]			1.00 (Reference)			
>115	0.14	0.121	1.15 (0.96–1.37)			
TPOAb						
[0, 34]			1.00 (Reference)			
>34	0.2	0.015	1.23 (1.04–1.44)			
HDL-c						
[1.16, 1.55]			1.00 (Reference)			1.00 (Reference)
<1.16	0.02	0.656	1.02 (0.93–1.13)	0.01	0.923	1.01 (0.89–1.14)
>1.55	−0.26	<0.001	0.77 (0.66–0.89)	−0.22	0.015	0.80 (0.68–0.96)
TC						
[2.8, 5.7]			1.00 (Reference)			1.00 (Reference)
<2.8	1.01	<0.001	2.75 (1.84–4.11)	0.41	0.111	1.50 (0.91–2.48)
>5.7	0.44	<0.001	1.56 (1.37–1.78)	0.35	<0.001	1.42 (1.21–1.65)
TG						
[0.29, 1.83]			1.00 (Reference)			1.00 (Reference)
>1.83	0.27	<0.001	1.30 (1.18–1.44)	0.1	0.105	1.11 (0.98–1.26)
GLU						
[3.9, 6.1]			1.00 (Reference)			
<3.9	−0.37	0.209	0.69 (0.39–1.23)			
>6.1	0.97	<0.001	2.64 (2.26–3.08)			
NE						
[1.8, 6.3]			1.00 (Reference)			1.00 (Reference)
<1.8	−0.16	0.267	0.85 (0.65–1.13)	0.04	0.794	1.04 (0.76–1.44)
>6.3	0.42	0.007	1.52 (1.12–2.05)	0.38	0.041	1.47 (1.02–2.12)
LYMPH						
[1.1, 3.2]			1.00 (Reference)			
<1.1	0.30	0.024	1.35 (1.04–1.75)			
>3.2	0.07	0.607	1.07 (0.83–1.38)			
MONO						
[0.10, 0.60]			1.00 (Reference)			1.00 (Reference)
>0.60	0.33	<0.001	1.39 (1.21–1.61)	0.14	0.133	1.15 (0.96–1.37)
RBC						
Male: [4.3, 5.8] Female:[3.8–5.1]			1.00 (Reference)			1.00 (Reference)
Male:<4.30 Female: <3.8	1.07	<0.001	2.93 (1.94–4.43)	0.67	0.007	1.95 (1.20–3.17)
Male: >5.80 Female: >5.1	−0.26	0.015	0.77 (0.63–0.95)	−0.17	0.161	0.84 (0.66–1.07)
PLT						
[125, 350]			1.00 (Reference)			
<125	0.4	0.173	1.49 (0.84–2.64)			
>350	−0.38	0.006	0.68 (0.52–0.90)			
HbA1C						
[4.8, 6.0]			1.00 (Reference)			1.00 (Reference)
<4.8	−1.13	0.001	0.32 (0.16–0.64)	−0.98	0.012	0.37 (0.17–0.81)
>6.0	1.06	<0.001	2.87 (2.56–3.23)	0.14	0.08	1.14 (0.98–1.33)

OR, odds ratio; CI, confidence interval. Univariate model: each variable entered alone.

Multivariate model: all variables with *P* < 0.10 in univariate analysis entered simultaneously.

Bold P values indicate statistical significance (*P* < 0.05).

### 5.4 Evaluation and presentation of the carotid plaque formation prediction model

As shown in Figure 2, the area under the receiver operating characteristic (ROC) curve (AUC) was 0.79 (0.78–0.80) for the development set and 0.80 (0.78–0.81) for the validation set, indicating good discriminatory ability of the model (Figure 2A) (22) pp. 177–180)). The nomogram is presented in Figure 2B. The decision curve analysis (DCA) curves for the development and validation sets exhibited nearly identical shapes and trends (Figures 2C,D). The Hosmer-Lemeshow test for the calibration curves yielded *P* = 0.468 for the development set and *P* = 0.091 for the validation set (Figures 2E,F), with both *P*-values > 0.05, suggesting no significant difference between the predicted probabilities and the observed probabilities, indicating good calibration [(22), pp. 157–162]. Furthermore, as shown in Table 7, the likelihood ratio test yielded *χ*^2^ = 1,961.8 for the development set and *χ*^2^ = 1,415.4 for the validation set, both with *P* < 0.0001, indicating statistical significance for both sets. The concordance index (C-index) was 0.79 (95% CI 0.78–0.80) for the development set and 0.80 (95% CI 0.78–0.81) for the validation set. The F1 scores were 0.738 vs. 0.729, and the Brier scores were 0.138 vs. 0.144, further demonstrating stable and good model discrimination (23, 24). Other core metrics also remained consistent, with details provided in Table 8.

**Table 7:** Model performance for predicting carotid plaque occurrence in the training and validation datasets.

Evaluation direction	Evaluation content	Training		Validation	
		Statistics	P	Statistics	P
Model verification	Likelihood ratio test	χ^2^: 1,961.8	<0.0001	χ^2^:1,415.4	<0.0001
Discrimination assessment (C-index)	(AUC, 95% CI)	0.79 (0.78–0.80)		0.80 (0.78–0.81)	
Calibration assessment	Hosmer-Lemeshow test.	χ^2^: 7.658	0.4676	χ^2^: 13.649	0.09138

AUC denotes the C-index. Hosmer-Lemeshow *P* > 0.05 indicates good calibration; the lower 95% CI bound of AUC > 0.5 confirms discrimination significantly better than chance.

**Table 8:** Confusion-matrix performance of the carotid-plaque prediction model in the training and validation.

Data	AUC (95% CI)	Accuracy (95%CI)	Sensitivity (95% CI)	Specificity (95% CI)	PPV (95% CI)	NPV (95% CI)	F1Score	Brier Score	cut off
Training	0.79 (0.78, 0.80)	0.73 (0.72, 0.74)	0.71 (0.69, 0.72)	0.76 (0.74, 0.77)	0.81 (0.79, 0.82)	0.64 (0.63, 0.66)	0.738	0.138	0.413
Validation	0.80 (0.78, 0.81)	0.73 (0.72, 0.74)	0.71 (0.70, 0.73)	0.76 (0.74, 0.77)	0.81 (0.80, 0.83)	0.64 (0.62, 0.66)	0.729	0.144	0.413

PPV, Positive Predictive Value; NPV, Negative Predictive Value.

### 5.5 Risk scoring table and risk assessment for carotid plaque formation

A risk scoring table for carotid plaque formation was established based on the logistic scoring method proposed by Sullivan et al. (25), as detailed in Table 1.

**Figure 4:**
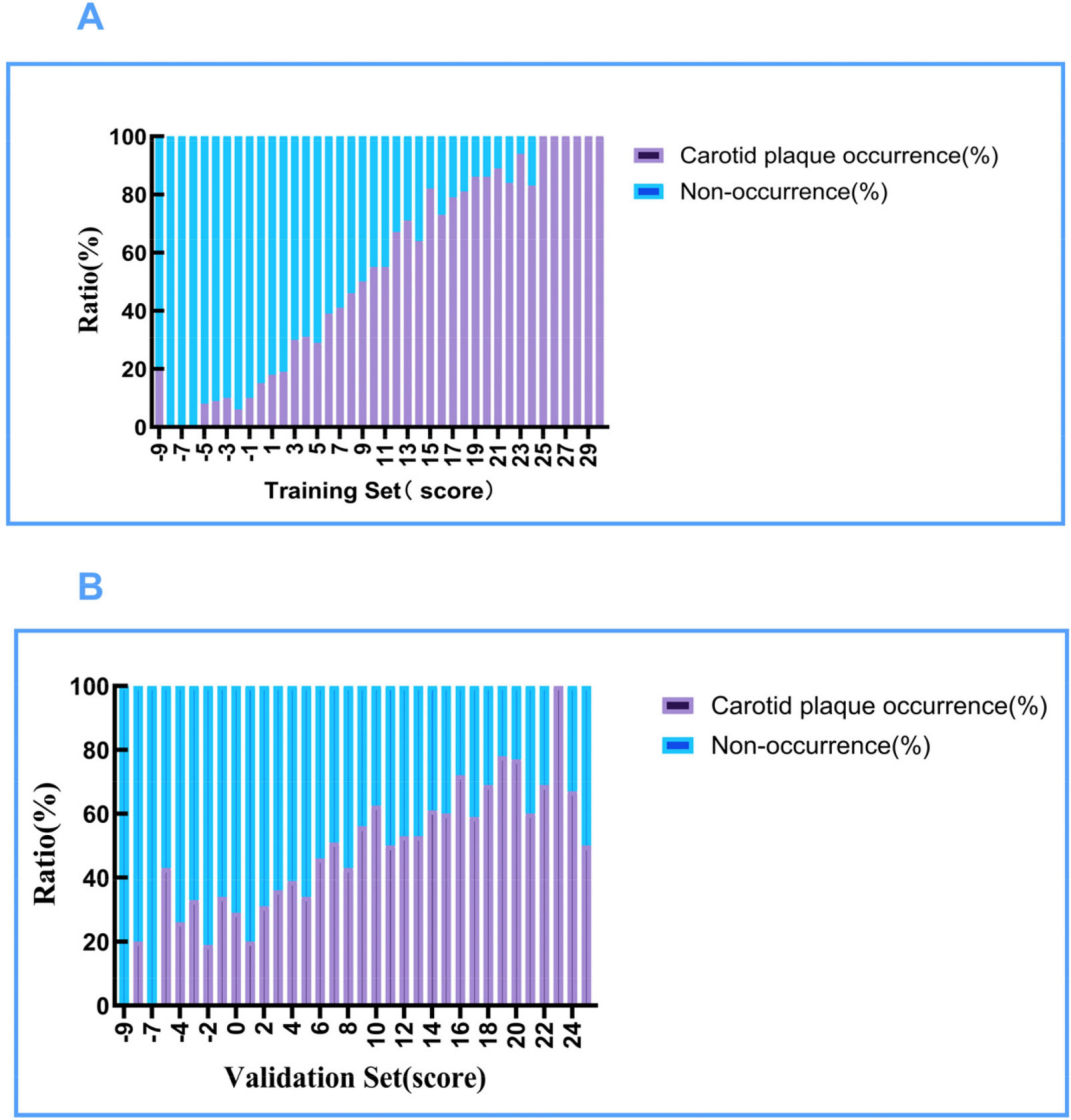
Stacked bar chart showing the proportion of carotid plaque occurrence (purple) and non-occurrence (blue) by risk-score category in the training set (upper panel) and validation set (lower panel).

In conjunction with Figure 4, the incidence of carotid plaque in both the development and validation set populations showed a gradual increasing trend as the assigned risk score increased. This gradient was further quantified and found to be significantly different in Table 9. The scoring model was subjected to binary logistic regression. Table 10 presents the incremental effect of individual risk score categories—low (−9 to 3 points), medium (4–6 points), and high (7–30 points)—on the detection risk of carotid plaque in both the development and validation sets. The P-values for all categories were <0.001, indicating that this scoring model possesses good risk discrimination ability in both population groups.

**Table 9:** Incidence of carotid artery plaque in the training and validation sets a cross risk-score Strata.

point	Training set			Validation set		
	Population	Plaque rate	Non-occurrence rate	Population	Plaque Rate	Non-occurrence rate
−9	5	20.00%	80.00%	1	0.00%	100.00%
−8	11	0.00%	100.00%	5	20.00%	80.00%
−7	2	0.00%	100.00%	2	0.00%	100.00%
−6	1	0.00%	100.00%	0	0.00%	100.00%
−5	12	8.33%	91.67%	21	42.86%	57.14%
−4	183	9.29%	90.71%	168	25.60%	74.40%
−3	381	10.24%	89.76%	377	32.63%	67.37%
−2	50	6.00%	94.00%	16	18.75%	81.25%
−1	108	10.19%	89.81%	114	34.21%	65.79%
0	1,140	15.09%	84.91%	1,033	29.04%	70.96%
1	124	17.74%	82.26%	59	20.34%	79.66%
2	877	19.04%	80.96%	756	30.56%	69.44%
3	115	29.57%	70.43%	44	36.36%	63.64%
4	178	31.46%	68.54%	109	38.53%	61.47%
5	250	29.20%	70.80%	194	34.02%	65.98%
6	335	38.51%	61.49%	303	46.20%	53.80%
7	113	40.71%	59.29%	43	51.16%	48.84%
8	162	45.68%	54.32%	98	42.86%	57.14%
9	622	50.00%	50.00%	407	55.77%	44.23%
10	143	54.55%	45.45%	48	62.50%	37.50%
11	771	54.60%	45.40%	464	50.00%	50.00%
12	297	67.00%	33.00%	129	52.71%	47.29%
13	308	71.43%	28.57%	114	52.63%	47.37%
14	263	63.88%	36.12%	128	60.94%	39.06%
15	225	81.78%	18.22%	78	60.26%	39.74%
16	159	72.96%	27.04%	54	72.22%	27.78%
17	121	78.51%	21.49%	54	59.26%	40.74%
18	146	80.82%	19.18%	54	68.52%	31.48%
19	79	86.08%	13.92%	18	77.78%	22.22%
20	92	85.87%	14.13%	35	77.14%	22.86%
21	37	89.19%	10.81%	5	60.00%	40.00%
22	51	84.31%	15.69%	13	69.23%	30.77%
23	18	94.44%	5.56%	2	100.00%	0.00%
24	29	82.76%	17.24%	9	66.67%	33.33%
25	7	100.00%	0.00%	0	0.00%	100.00%
26	12	100.00%	0.00%	2	50.00%	50.00%
27	3	100.00%	0.00%	0	0.00%	0.00%
28	2	100.00%	0.00%	0	0.00%	0.00%
29	1	100.00%	0.00%	0	0.00%	0.00%
30	1	100.00%	0.00%	0	0.00%	0.00%
−9 to 3	3,009	16.00%	84.00%	2,596	30.00%	70.00%
4–6	763	34.00%	66.00%	606	41.00%	59.00%
7–30	3,662	63.00%	37.00%	1,755	56.00%	44.00%

The table summarizes the incidence of carotid artery plaque in the training (*n* = 7,434) and validation (*n* = 4,957) sets across risk-score strata ranging from –9 to +30 points.“Population” denotes the number of participants within each score interval; “Plaque Rate” indicates the proportion with ultrasound-detected carotid plaque; “Non-occurrence Rate” represents the proportion without detectable plaque. The bottom three rows merge the scores into low-risk (–9 to 3), intermediate-risk (4–6) and high-risk (7–30) categories, presenting the overall plaque prevalence for each tier.

**Table 10:** Incidence and absence rates of carotid artery plaque at different risk scores.

Variables	Training Set					Validation Set				
	Β	S.E	Z	P	OR (95%CI)	β	S.E	Z	P	OR (95%CI)
−9 to 3					1.00 (Reference)					1.00 (Reference)
4–6	0.48	0.09	5.19	<0.001	1.62 (1.35–1.95)	1.02	0.09	11.17	<0.001	2.78 (2.32–3.33)
7–30	1.08	0.06	16.71	<0.001	2.93 (2.59–3.33)	2.24	0.06	36.8	<0.001	9.41 (8.35–10.60)

Carotid plaque risk across aggregated score strata. β, standard error (SE), Wald Z and two-sided P are derived from logistic regression with the low-risk stratum (–9 to 3 points) as reference. Odds ratios (OR) and 95% confidence intervals (CI) quantify the incremental likelihood of plaque detection in the 4–6 and 7–30 point categories for the training (*n* = 7 434) and validation (*n* = 4,957) sets, respectively.

## 6 Discussion

This study developed a regression prediction model for risk factors associated with carotid plaque formation, validated its performance, and subsequently established a risk scoring table. The aim is to assist clinicians in rapidly screening high-risk populations and implementing precise stratified management for patients with existing plaques.

Our model employed lasso regression and multivariate logistic regression analysis to identify 12 potential risk factors: female sex, age, smoking, hypertension, coronary heart disease, diabetes mellitus, elevated TSH, elevated HDL-C, elevated TC, elevated NE, decreased RBC, and decreased HbA1c. The model was evaluated using the area under the curve (AUC) of the receiver operating characteristic curve, calibration curves, and decision curve analysis (DCA). The results demonstrated its high discriminative ability, reliability, and clinical utility. Based on these findings, we assigned scores to individuals in the development and validation sets according to their plaque incidence. A three-tiered risk stratification system (−9 to −3, 4–6, 7–30) was adopted, with its boundaries simultaneously meeting the three gold standards proposed by PROBAST (26). This strategy of initial stratification followed by refinement ensures that high-risk individuals are not missed while avoiding excessive testing for low-risk populations.

Our findings indicate that age is the primary risk factor for carotid plaque development. The risk scoring table from this study assigns a single-item score of up to 15 points to the >60 years age group, suggesting that advanced age may be an independent driving factor for plaque formation. Firstly, studies have confirmed that aging is accompanied by a 30%–40% decrease in hepatic LDL receptor expression and a 1.6-fold prolongation of plasma LDL-C retention time, leading to prolonged vascular exposure despite normal lipid concentrations (27). Secondly, vascular endothelial repair capacity declines with age, with repair rates in elderly individuals reduced by over 50%, prolonging the exposure time of the lipid core and pushing plaque formation into an irreversible stage (28). Therefore, lifestyle modification should be intensified in the elderly population to partially offset the dual disadvantages of metabolic and repair deficits associated with aging.

Notably, this study is the first to incorporate thyroid function indicators to explore their impact on carotid plaque formation and their contribution to the associated risk score. Thyroid hormones regulate heart rate, blood pressure, lipid metabolism, and vascular endothelial function. Even subclinical hypothyroidism can elevate LDL and promote atherosclerosis (29, 30). However, no large-scale studies have yet integrated thyroid function into an intuitive plaque risk scoring system. Our results demonstrate that elevated TSH is a risk factor for carotid plaque formation and is positively correlated with its occurrence. Several recent cross-sectional studies also indicate a positive correlation between TSH levels and both carotid plaque prevalence and severity, identifying TSH as a predictor of plaque severity (31, 32). The underlying mechanism may involve persistent TSH stimulation inducing a low-grade inflammatory state, characterized by an increase in peripheral activated neutrophil count, which is reflected in our scoring table by assigning 2 points for elevated NE. These activated neutrophils can directly attack the vascular wall and recruit monocytes through various mechanisms, including the release of IL-1β, ROS, and the formation of neutrophil extracellular traps (NETs), ultimately promoting plaque initiation and progression (33, 34). The assignment of 2 points for elevated TC in the table further suggests that TSH influences plaque formation not only through inflammatory pathways but also via lipid metabolism disorders. Elevated TSH leads to increased TC and LDL-C levels, delays cholesterol clearance, and reduces HDL-C synthesis (35). Li Z et al. further demonstrated a positive correlation between elevated TSH and both TC and LDL-C, with a more pronounced effect in overweight populations, confirming that TSH affects cardiovascular risk through lipid pathways (36). In our scoring table, TSH is assigned a weight of 1 point, which does not underestimate its importance but rather aims to achieve a quantifiable link between endocrine function and vascular pathology.

Furthermore, this study found that a red blood cell (RBC) count below the normal lower limit may be a strong risk factor for atherosclerosis, and we assigned 4 points for this parameter. Studies suggest that decreased RBC count promotes atherosclerosis via the axis of “reduced NO bioavailability leading to impaired endothelium-dependent vasodilation, thereby creating a pro-inflammatory and pro-adhesive environment that accelerates atherosclerosis initiation” (37). Our findings corroborate this view. This suggests that actively correcting anemia in clinical practice could potentially serve as a novel primary or secondary prevention strategy for cardiovascular disease. Clinicians should maintain a higher level of vigilance for individuals with low RBC counts and consider it a potential signal of cardiovascular risk. HbA1c levels below the normal range exhibited a strong protective effect, indicating that a lower glycation state may confer vascular protection by reducing the direct toxic damage and oxidative stress inflicted on tissues and cells by chronic hyperglycemia (38). Smoking, hypertension, coronary heart disease, and diabetes mellitus were identified as risk factors, which is highly consistent with the existing body of knowledge. Compared to non-smokers, smoking not only causes endothelial cell injury but also leads to lipid metabolism disorders, further promoting atherosclerosis. Hypertension also predisposes to endothelial cell injury. Based on the influence of hemodynamics and shear stress on vascular endothelial function (39), faster blood flow causes more significant damage to endothelial cells at vascular bifurcations, increasing opportunities for LDL-C to enter the endothelium and subsequently leading to atherosclerosis. As a potential lesion of atherosclerotic cardiovascular disease (ASCVD), the presence of carotid plaque can predict coronary heart disease risk (40). On one hand, patients with coronary heart disease should be proactively screened for carotid plaque to assess pan-vascular atherosclerotic burden. On the other hand, individuals with positive carotid plaque findings require intensified primary prevention for ASCVD. Additionally, female sex and elevated HDL-C were identified as protective factors in our model. Before menopause, endogenously secreted estrogen in women can resist atherosclerosis through mechanisms such as improving the lipid profile, exerting antioxidant effects, and providing anti-inflammatory benefits (41, 42). HDL-C is a well-known protective lipid, reducing atherosclerosis formation through anti-inflammatory, antithrombotic, and anti-apoptotic actions (43). However, recent studies have found that under conditions of chronic inflammation or oxidative stress, HDL particles can become oxidized or glycated, losing their protective functions and even converting to a pro-inflammatory state (44). Therefore, a high HDL-C level does not always equate to strong protection. This highlights the need to focus not only on the quantity of HDL-C but also on its functional relationship with atherosclerosis formation. Future research could further investigate HDL-C subclasses and their correlation with inflammatory markers to better understand their impact on atherosclerosis.

In summary, this scoring table can assist clinicians in screening high-risk populations within 30 seconds in outpatient settings and enable precise stratified management for patients with existing plaque. It provides an operational and scalable tool for the early warning and individualized intervention of carotid plaque and even pan-vascular atherosclerosis. However, the retrospective nature of this study precludes causal inference. Future prospective cohort studies are needed to evaluate the predictive ability of this score for incident plaque.

## 7 Conclusion

In conclusion, this study identified 12 independent risk factors for carotid plaque and developed a risk stratification scoring table. This scoring table aids frontline clinicians in identifying high-risk populations, avoiding excessive examination of low-risk individuals, and potentially improving patient prognosis while reducing healthcare costs. Future multi-center prospective cohort studies are warranted to further optimize carotid plaque prediction and intervention strategies.

## Data Availability

The raw data supporting the conclusions of this article will be made available by the authors, without undue reservation.
